# Field Sandbur (*Cenchrus pauciflorus*) Seeds in the Same Bur Respond Differently to Temperature and Water Potential in Relation to Germination in a Semi-Arid Environment, China

**DOI:** 10.1371/journal.pone.0168394

**Published:** 2016-12-19

**Authors:** Zhixin Zhang, Xun Tian, Yuguang Bai, Huifang Liu, Xueli Niu, Zhiwei Wang, Qian Wang

**Affiliations:** 1 The State Key Laboratory of Grassland Agro-Ecosystem, Lanzhou University, Lanzhou, Gansu, China; 2 College of Pastoral Agriculture Science and Technology, Lanzhou University, Lanzhou, Gansu, China; 3 College of Life Science, Inner Mongolia University for Nationalities, Tongliao, Inner Mongolia, China; 4 College of Agriculture and Bioresources, University of Saskatchewan, Saskatoon, Canada; 5 Guizhou Institue of Prataculture, Guizhou Academy of Agricultrual Sciences, Guiyang, Guizhou, China; University of Illinois at Urbana-Champaign, UNITED STATES

## Abstract

The success of a biological invasion relies on the environment and is closely linked to factors such as water and temperature. Invasive plant species display different seed characteristics, including shape. Field sandbur (*Cenchrus pauciflorus*) is a globally widespread invasive species capable of adapting to broad environmental conditions. However, its germination response to water and temperature still remains unclear. *C*. *pauciflorus* contains two seeds in the same bur that differ in size: big seeds (M) and small seeds (P). Separate greenhouse experiments were conducted under different temperature regimes (0/10°C, 5/15°C, 10/20°C, 15/25°C, 18/28°C, 20/30°C and 25/35°C) and water potentials (-1.50Mpa, -1.00Mpa, -0.75Mpa, -0.50Mpa, -0.25Mpa and 0Mpa) for M and P seeds. The results support the hypothesis that germination of *C*. *pauciflorus* is significantly influenced by seed type, temperature and water potential. M and P seeds responded differently to varied alternative temperatures and water potentials. However, M and P seeds were more sensitive to water potential than to temperature. Optimal conditions for M and P seed germination were measured at 25/35°C (night temperature/day temperature) and 20/30°C, respectively. In contrast, the highest germination rate was observed for the 0Mpa of the water potential treatment. Additionally, base temperature (T_base_) and base water potential (W_base_) were lower for M (7.7°C, -1.11Mpa at 10/20°C, and -1.07Mpa at 20/30°C) than for P (9.4°C, -0.92Mpa at 10/20°C, and -0.52Mpa at 20/30°C). These different germination strategies of M and P seeds with respect to temperature and water potential increased overall plant propagation. These results indicate that tropical and subtropical regions water potentials beyond -0.50Mpa (10/20°C) or -1.00Mpa (20/30°C) face a potential risk of *C*. *pauciflorus* invasion.

## Introduction

Due to climate change, ecosystems are facing an increasing risk of exotic plants invasion [1, 2]. More than 270 invasive plant species have been identified in China, most of which are annual herbs [3]. They have caused huge regional economic loss and are threatening the stability of ecological environment, biology diversity and human health. An estimated US$ 14.45 billion of annual economic loss is caused as a results of invasive alien species in China[4].

Invasive plant species show strong adaptability and anti-adversity in a range of environments beyond their native habitats. This has been considered an important trait for successful establishment in new habitats [5]. Plants develop rapidly to compete with native species for an ecological niche in a favorable environment. They show various survival strategies under harsh conditions, e.g. early seed germination, consistently high and rapid germination rates, short time requirements for seed stratification and production of high seedling biomass [5–9]. These characteristics facilitate the spread of invasive plants to new environments.

In the agro-ecosystem, germination is a key step in the process of successful population establishment for any weed. Each species has its own requirements for germination in an unpredictable environment and a strategy to maximize survival [10]. The different environmental sensitivities are predominantly caused by variations of numerous factors involved. Temperature and water availability are two of the primary environmental factors that control seed germination rate, final percentage germination, and seedling establishment throughout species [11–16]. Polyethylene glycol (PEG) 6000 solutions are frequently used for setting water potential in a wide range without affecting seed germination [17]. Improved understanding of biology and mechanisms of seed germination under different temperature and water osmotic potentials is critical to build viable models and evaluate potential risks of plant invasion. How temperature and water potential affects invasive plant species such as *Ceratocarpus arenarius*, *Piper aduncum* and *Tithonia diversifolia* has been reported. These reports have identified a system for invasion risk estimation, illuminating the importance of adaptation and possible mechanisms of invasion [18–20]. In these reports, base temperature and water potential for germination refer to the temperature or water potential below which seed germination cannot occur for any given species. These are key parameters for predicting and modelling the periods during which germination is possible and to decipher how they vary with species [21].

Variation in seed size and shape is an important phenomenon of heteromorphism. This characteristic was also deemed a possible reason for invasiveness in favor of adapting to a diverse environment [22, 23]. Different seed type and size result in variation of dormancy, germination, dispersal strategy and competitiveness [22, 23, 24]. Increased size of seeds improves germination rate, seedling survival, shortens germination duration, as well as enhances seedling growth compared to smaller seeds due to more nutrient investment and higher quality thereof [25]. Goatgrass (*Aegilops cylindrical*) produces larger seed in the second or third positioned floret, and smaller seed in the primary positioned floret, larger seed consistently germinates first [26].

Field sandbur (*Cenchrus pauciflorus*) (S1 Fig), is known under many names (e.g. *Cenchrus echinatus*, *Cenchrus incertus*, *Cenchrus parviceps*, *Cenchrus spinifex*, grass bur, coastal sandbur, Tribulus terrestris grass or nettles [27]) and is a plant native to the South American and African continent and is widely distributed throughout tropical and subtropical environments [28]. It is especially well adapted to dry and sandy soils, but is also distributed in other types of soils [28, 29]. The dispersal of sandbur mainly relies on its spiny bur [28], which can easily be found near roads, farm fields, beaches and natural pastures [30]. Currently, the plant has become a widespread invasive species in many countries of the world [31–34]. In China, it has been found in Liaoning, Inner Mongolia, and the Jilin provinces of China since a first report in Liaoning [30], and the area of its distribution increases every year. Each raceme produces more than 70 spiny burs, containing 1–3 seeds (typically two different seed sizes) that easily attach to animal hair or clothes [30]. This has an enormous negative impact on sustainable development of agriculture and husbandry, causing injuries to human and animal skin, contaminating feeds and reducing palatability for animal consumption [28]. The plant furthermore competes for light, nutrition, water and other resources with native species, inhibits growth and lowers the quality of other plants [30]. *C*. *pauciflorus* cannot be manually weeded due to the spines of the bur, making it a noxious weed for farmers. Attempts to reduce the invasion of *C*. *pauciflorus* are made via mowing and adjusting row spacing [35, 36]. However, such methods are only applicable in a small scale and the effect is limited, leaving no effective way to control and remove *C*. *pauciflorus* from most of the invaded fields. To understand why *C*. *pauciflorus* is so prolific, it is crucial and urgent to gain a better understanding of how seed germination and emergence responds to temperature and water potential.

However, current knowledge of how temperature and water potential affect germination of the two different seeds types of *C*. *pauciflorus* is limited. Therefore, the objectives of this study were to: 1) Evaluate the effect of temperature and water potential on the germination rate of *C*. *pauciflorus* seeds (M and P) in the same bur; 2) Quantify the base thermal time and base water potential for successful germination of *C*. *pauciflorus* seeds.

## Materials and Methods

### Seed collection

**Fig 1:**
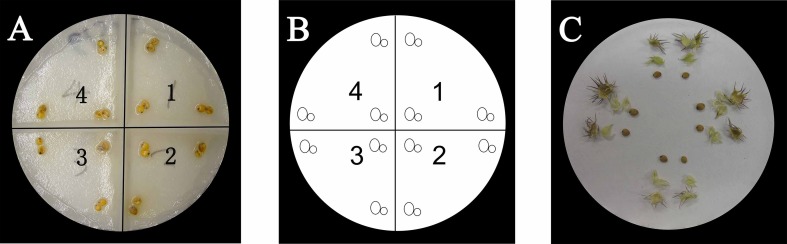
**Layout of M and P seeds of *C*. *pauciflorus* in petri-dish real (A) and model (B) in the experiment. Bur structure of *C*. *pauciflorus* with separated bur coat, seed coat and M, P seed (C)**.

Mature burs of *C*. *pauciflorus* were collected from Horqin sandy land in August 2010 (43.52° N 122.32° E) and stored in paper bags below 4°C in darkness before commencement of experiment (The owner of the land gave permission to conduct the study on this site). The mean bur dry weight per 100 burs was 3809 ± 716 g. All seeds were less than eight months old when the experiment was conducted. In each bur, there are two distinct seeds in size and mass due to the cryptic heteromorphism, which can be recognized visually. We defined the big seeds (with lengths of 2.5–3.5 mm, widths of 1.5–2.9 mm, and thicknesses of 0.9–2.0 mm) as Mango-type (M) and small seeds (with lengths of 1.4–3.0 mm, widths of 1.5–2.8 mm, and thicknesses of 0.7–1.7 mm) as Plum-type (P) based on their size and shape (Fig 1) (C). Measurement and statistical results of M and P seeds were provided in S2 Fig and S1 Table. The average weights of M and P were 8.2 ± 2.2 mg and 5.6 ± 1.5 mg per seed, respectively. Seed moisture ranged from 3% to 5%, and no significant difference of seed moisture was detected between M and P. Both the two type seeds were prepared one week before the experiment by manually dissecting M and P seeds from each bur. After separation of one bur was done, M and P seeds were immediately placed in two containers, one was used to collect M seeds and the other was used to collect P seeds.

### Temperature and water potential treatments

Two experiments were carried out at different levels of temperature and water stress. Seeds in experiment 1 were set at different alternating (night time/day time) temperatures of 0/10°C, 5/15°C, 10/20°C, 15/25°C, 18/28°C, 20/30°C and 25/35°C to simulate field temperatures in different months. Seeds in experiment 2 were placed on a polyethylene glycol solution (PEG_6000_) at -1.50 Mpa, -1.00 Mpa, -0.75 Mpa, -0.50 Mpa, -0.25 Mpa and 0 Mpa at 10/20°C and 20/30°C. Control refers to 0Mpa with ample water is available. All treatments were subjected to a 12 h night and 12 h day cycle. The temperatures in experiment 2 were selected based on the optimal temperatures found in experiment 1.

### Germination tests

Twelve seeds were placed into one Petri dish each (diameter of 100 mm), fitted with a double layer of humid filter paper. Six replications were conducted per treatment in a completely randomized design and dampened with deionized water (water potential: 0Mpa) to evaluate germination. All Petri dishes were kept in sealed plastic bags to prevent the seeds from desiccating. The filter paper used in the experiment was folded in a cross-shape ridge for easy data recording (Fig 1A and 1B). Prior to each experiment, seeds were sterilized with 2% sodium hypochlorite solution for 30–60 s, and then washed five times with distilled water. The treatments were incubated in environmentally controlled growth chambers (CMP3244 Conviron, Canada) with constant relative humidity (75%). All treatments were periodically re-irrigated to avoid desiccation. Germination was identified as the protrusion of the radicle (3 mm) and continuously monitored every 2 h at the beginning of the incubation period, or every 12 h as germination percentage approached the plateau (around 80%) until either all seeds had germinated or no additional germination occurred for five consecutive days. Germination percentage was calculated for a viable fraction of the seed population. Sigmoidal curves were fitted with the mean value to obtain the time needed to achieve 50% of the maximum germination of viable seeds (T_50_). The average temperature was used for alternative temperature treatments. The germination rate was calculated as 1/T_50_. Data outside the linear range effect of temperature (or water potential) extremes were dropped [37].

### Data analysis

All data are expressed as mean values with standard error (in bracket) in tables and the mean values in figures. Treatment effects of accumulated germination rates from all experiments were separately analyzed using one-way (for temperature or water potential treatments), two-way (for main effects and the interaction between temperature and water potential) or three-way (for main effects and interaction between seed type, temperature, water potential) analysis of variance (ANOVA) and a significance level of *P* < 0.05 via the ANOVA packages of SPSS 22.0 (IBM Corporation, USA). Data of all germination percentages were subjected to an arcsine transformation subsequent to ANOVA to ensure homogeneity of variance (all figures depict non-transformed data). Mean comparisons were performed via least significant difference (LSD) to determine whether observed differences among means were significant between treatments and within each temperature, water potential and seed type (*P* < 0.05). To avoid distortion of the final results, outlier values were dropped from the analysis. Outliers were defined via a comparison of the differences between values and the 3.0×interquartile range [38].

## Results

### Effects of temperature on germination of M and P seeds

**Fig 2:**
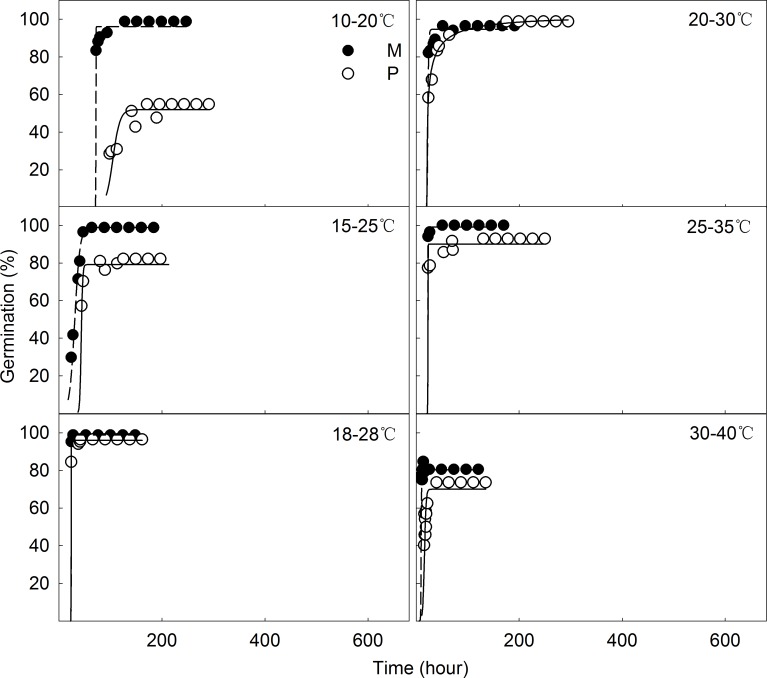
Cumulative M (solid dot) and P seeds (hollow dot) germination time-courses of *C*. *pauciflorus* under seven temperature regimes.

Temperature significantly impacted the mean final germination time (T_FG_) and final germination percentage (FGP) of *C*. *pauciflorus* (*P* < 0.05). Specifically, each increment in temperature shortened T_FG_ of both M and P seeds, although we only observed a significant difference in M seeds at 10/20°C. Final germination percentage was relatively stable among different temperature treatments for M. However, germination percentage of P seeds increased with temperature until 30/40°C, beyond which it slightly declined. A significant increment could be observed between 10/20°C and 15/25°C, as well as between both treatments and other treatments (18/28°C, 20/30°C, 25/35°C, and 30/40°C) for P, while no significant differences were found in M. Therefore, treatments from 10/20°C to 30/40°C and from 18/28°C to 30/40°C were revealed as an optimal temperature for the germination of M and P seeds of *C*. *pauciflorus*, respectively. The maximum value of germination for M seeds was observed in the 25/35°C treatment, while P seeds had a maximal germination percentage at 20/30°C (Table 1, Fig 2).

**Table 1:** Effects of temperature and seed type on final germination percentage (FGP) and mean final germination time (T_FG_) of field sandbur.

Temperature (°C)	T_FG_ (hours)						FGP (%)					
	M			P			M			P		
5/15	-	-	-	-	-	-	-	-	-	-	-	-
10/20	115.3	(11.4)	Aa	217.4	(40.9)	Ab	96.4	(1.7)	Aa	52.4	(5.0)	Cb
15/25	47.0	(0.0)	BCa	144.4	(33.0)	ABb	97.2	(1.7)	Aa	76.6	(1.7)	Bb
18/28	44.0	(0.0)	BCa	144.1	(6.2)	ABb	98.6	(1.4)	Aa	96.4	(1.7)	Aa
20/30	60.1	(13.4)	Ba	145.3	(28.9)	ABb	95.2	(2.5)	Aa	97.6	(1.5)	Aa
25/35	34.8	(8.2)	BCa	111.9	(25.3)	ABb	100	(0.0)	Aa	92.9	(2.8)	Ab
30/40	17.2	(1.5)	Ca	62.8	(23.4)	Ba	98.3	(1.7)	Aa	91.7	(2.6)	Aa
mean	52.7	(6.5)	a	122.8	(14.5)	b	97.5	(0.7)	a	84.7	(3.0)	b

Note: Capital letters in the same column represent significant differences under different temperature treatments at *P* < 0.05. Lower case letters represent significant differences between M and P seeds at *P* < 0.05.

Differences in T_FG_ of M and P seeds were found in almost all treatments, while these were significant for 10/20°C, 15/25°C, and 25/35°C for FGP. Compared to P, T_FG_ was shortened by 45 hours on average and FGP improved by 15% for M seeds (Table 1, Fig 2).

Temperature and seed type substantially affected T_FG_ and FGP of *C*. *pauciflorus* (*P* < 0.001), whereas M and P seeds performed similarly under varied alternative temperature treatments towards T_FG_. A strong interaction was observed between temperature and seed type (*P* < 0.001), indicating that M and P seeds showed a different response among the temperature treatments at FGP (Table 2).

**Table 2:** Two-way ANOVA analysis of temperature, seed type and their interaction on T_FG_ and FGP of field sandbur.

Indicator	Source	df	F	Sig.	Sig.
T_FG_	Temperature	5	10.618	***	< .001
	Seed type	1	32.390	***	< .001
	Temperature * Seed type	1	1.114	n.s.	0.362
FGP	temperature	5	29.043	***	< .001
	Seed type	1	82.019	***	< .001
	Temperature * Seed type	1	25.962	***	< .001

*** indicates significantly different at *P*<0.001.

n.s. indicates no significantly different at P<0.05.

### Effects of water potential and temperature on the germination of M and P seeds

**Fig 3:**
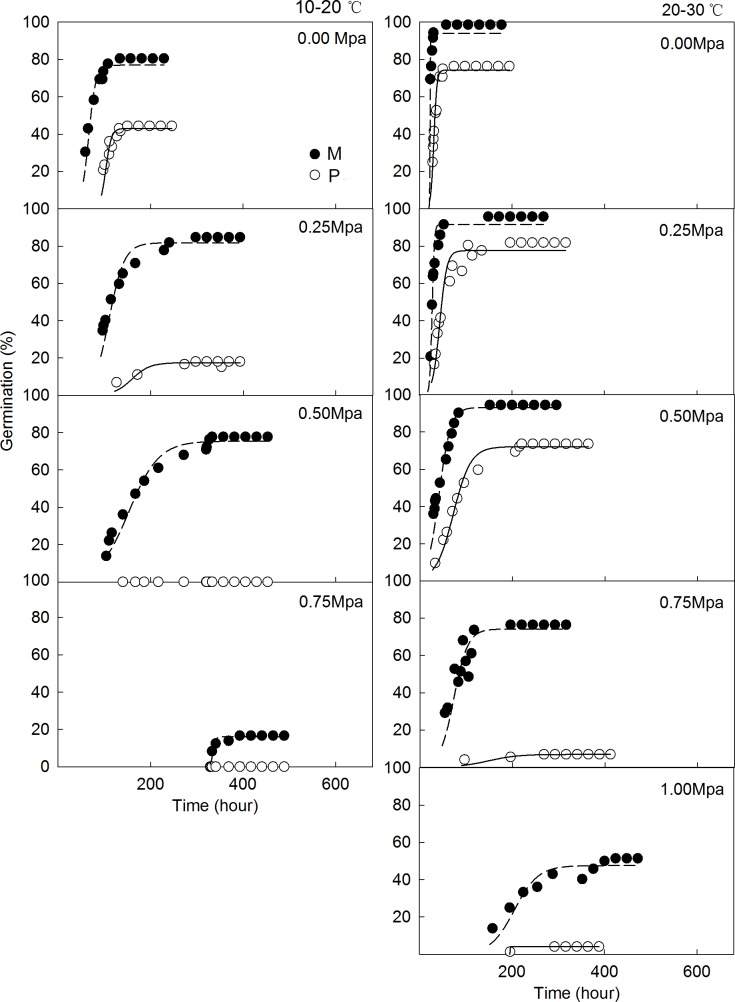
Cumulative M (solid dot) and P seeds (hollow dot) germination time-courses of *C*. *pauciflorus* under two temperatures and six osmotic potential regimes. Different symbols represent the observed germination percentages against time at different osmotic potentials. None of seeds germinated at 1.00 and 1.50Mpa at 10–20°C under 12-hour dark and 12-hour light condition.

Increasing water potentials under both 10/20°C and 20/30°C temperatures prolonged T_FG_ and reduced FGP for M and P seeds. Under 10/20°C, T_FG_ and FGP changed from 129.2 to 351 hours, and from 96.7% to 12.5% for M seeds, and from 136.0 to 311.6 hours, and 58.3% to 18.1% for P seeds, respectively. Similarly, for 20/30°C, T_FG_ and FGP ranged from 37.0 to 316.0 hours, and 97.2% to 47.2% for M seeds, and from 51.0 to 292 hours, and 90.0% to 5.0% for P seeds. Moreover, 20/30°C, treatments with equal water potential generally took less time and maintained a higher germination percentage compared to the 10/20°C treatment (*P* < 0.05) (Table 3, Fig 3).

**Table 3:** Effects of water potential of PEG_6000_ solution and temperature on final germination percentage FGP and T_FG_ of field sandbur M and P seeds.

Temperature (°C)	Water potential (Mpa)	T_FG_ (hours)						FGP (%)					
		M			P			M			P		
10/20	Control	129.2	(19.4)	Aa	136.0	(14.0)	Aa	96.7	(2.0)	Aa	58.3	(9.6)	Ab
	-0.25	248.5	(20.0)	Ba	311.6	(66.1)	Ba	81.9	(4.0)	Aa	18.1	(6.3)	Bb
	-0.50	302.5	(41.2)	Ba	-	-	-	70.8	(5.4)	Aa	-	-	-
	-0.75	351.0	(31.6)	Ba	-	-	-	12.5	(2.4)	Ba	-	-	-
	-1.00	-	-	-	-	-	-	-	-		-	-	-
	-1.50	-	-	-	-	-	-	-	-		-	-	-
	mean	244.0	(22.6)	a	245.8	(51.1)	a	42.2	(7.6)	a	8.9	(3.4)	b
20/30	Control	37.0	(4.4)	Aa	51.0	(2.5)	Ab	97.2	(1.8)	Aa	90.0	(1.7)	Aa
	-0.25	54.7	(4.8)	Aa	169.2	(37.4)	ABb	93.1	(1.4)	Aa	81.9	(5.9)	Ab
	-0.50	159.5	(38.3)	Ba	256.2	(28.0)	Ba	94.5	(4.1)	ABa	73.6	(5.0)	Bb
	-0.75	189.2	(30.3)	Ba	221.7	(58.7)	Ba	75.0	(8.6)	Ba	6.9	(4.0)	Bb
	-1.00	316.0	(39.2)	Ba	292.0	(0.0)	Ba	47.2	(3.5)	Ca	5.0	(2.0)	Bb
	-1.50	-	-		-	-	-	-	-	-	-	-	-
	mean	151.3	(22.1)	a	179.2	(23.0)	a	69.8	(6.0)	a	42.6	(7.0)	b

Note: Capital letter means significant difference among water potential treatments at P<0.05, lowercase letter indicates significant difference between M and P seed at P< 0.05. The dash line indicates no statistical analysis was conducted due to lack of germination data in the treatment or recorded data was extreme value.

For both temperatures, control T_FG_ was significantly shorter compared to other germinated treatments with one except for the -0.25Mpa treatment at 20/30°C (*P* < 0.05). No significant variation was observed for different water potentials in the other germination treatments. However, FGP of M seeds at -0.75Mpa (12.5%) and P seeds at -0.25Mpa (18.1%) varied significantly compared to the treatments with lower water potential and the 0Mpa treatment at 10/20°C (*P* < 0.05). Similarly, for M seeds, FGP at 20/30°C significantly differed between treatments of -0.75Mpa and -1.00Mpa, FGP was also significantly lower for -1.00Mpa compared to other treatments (control, -0.25Mpa, -0.50Mpa, and -0.75Mpa). For P seeds, FGP differed significantly at equal temperatures between -0.50Mpa, -0.75Mpa, and -1.00Mpa treatments and control, and -0.25Mpa treatment (*P* < 0.05) (Table 3, Fig 3).

Significant (*P* < 0.05) differences for T_FG_ were only observed between M and P seeds for 0Mpa and -0.25Mpa treatments at 20/30°C among all levels of water potentials. In contrast, significant differences of FGP were detected for all germinated treatments under 10/20°C and 20/30°C conditions (*P* < 0.05). Furthermore, germination activity of M and P seeds stopped at -1.00Mpa and -0.50Mpa for the 10/20°C treatment, and both disappeared at -1.50Mpa for 20/30°C. The highest final germination percentage occurred in the control treatment for both temperatures for M and P seeds. On average, M seeds germinated quicker (2, 28 hours) and with higher germination rate (33%, 27%) at 10/20°C and 20/30°C treatments and compared to P seeds (Table 3).

Temperature and water potential were significantly affected T_FG_ and FGP. Seed type did not show any significant differences in T_FG_, but in FGP. No interaction was observed at T_FG_, while, strong interactions of temperature and water potential, water potential and seed type, as well as temperature, water potential and seed type were found in FGP. These results revealed that, temperature, water potential and seed type play important roles for the germination of *C*. *pauciflorus* (Table 4).

**Table 4:** Effects of temperature, water potential, seed type and their interactions on T_FG_ and FGP of field sandbur.

Indicator	Source	df	F	sig	Sig.
T_FG_	temperature	1	40.730	***	<0.001
	water potential	4	19.618	***	<0.001
	seed type	1	2.901	n.s.	0.093
	temperature * water potential	3	1.011	n.s.	0.394
	temperature * seed type	1	0.395	n.s.	0.532
	water potential * seed type	4	1.238	n.s.	0.304
	temperature * water potential * seed type	1	0.225	n.s.	0.637
FGP	temperature	1	291.335	***	<0.001
	water potential	5	301.227	***	<0.001
	seed type	1	307.395	***	<0.001
	temperature * water potential	5	19.644	***	<0.001
	temperature * seed type	1	3.560	n.s.	0.648
	water potential * seed type	5	19.009	***	<0.001
	temperature * water potential * seed type	5	36.928	***	<0.001

*** indicates significantly different at *P*<0.001

n.s. indicates no significantly different at P<0.05.

### Relationship between temperature and water potential with 1/T_50_ for M and P seeds

**Fig 4:**
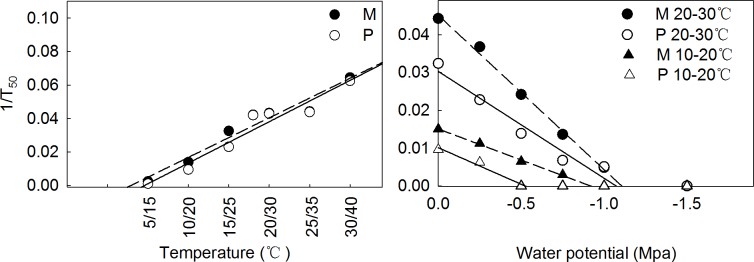
Linear relationship of temperature (calculated from average value of alternative temperature) and 1/T_50_ (left), water potential and 1/T_50_ (right) under 10/20°C, 20/30°C environment of M (solid dot) and P seed (hollow dot) of *C*. *pauciflorus*. Linear regression functions of temperature were *y* = -0.0185 + 0.0024*x* (*R*^*2*^ = 0.944) (M seed), *y* = -0.0235 + 0.0025*x* (*R*^*2*^ = 0.970) (P seed), respectively. Linear regression functions under different water potential were *y* = 0.0151 + 0.0164*x* (*R*^*2*^ = 0.999) (M seed at 10/20°C), *y* = 0.0101 + 0.0193*x* (*R*^*2*^ = 0.986) (P seed at 10/20°C), and under 20/30°C were *y* = 0.0451 + 0.0408*x* (*R*^*2*^ = 0.997) (M seed at 20/30°C), *y* = 0.0303 + 0.0282*x* (*R*^*2*^ = 0.976) (P seed at 20/30°C), respectively. Germination rate (y) calculated as the inverse of T_50_, that is, the number of days necessary to reach 50% of the final germination proportion.

Linear regression was conducted to determine the base temperature (T_base_) and base water potential (W_base_) of M and P seeds under greenhouse conditions. The effect of temperature could be described by functions of *y* = -0.0185 + 0.0024*x* (R^2^ = 0.944) (M seed), *y* = -0.0235 + 0.0025*x* (R^2^ = 0.970) (P seed), respectively. In contrast, the influence of water potential at 10/20°C followed functions of *y* = 0.0151 + 0.0164*x* (R^2^ = 0.999) (M seed), *y* = 0.0101 + 0.0193*x* (R^2^ = 0.986) (P seed). Under 20/30°C, the influences of water potential were *y* = 0.0451 + 0.0408*x* (R^2^ = 0.997) (M seed), *y* = 0.0303 +0.0282*x* (R^2^ = 0.976) (P seed), respectively. The results calculated from the *x* interceptions revealed T_base_ of M and P seeds to be 7.7 and 9.4°C, respectively. However, W_base_ at 10/20°C and 20/30°C treatments were -1.11Mpa and -0.92 Mpa for M seeds and -1.07Mpa and -0.52 Mpa for P seeds (Fig 4).

## Discussion

Germination is one of the most important events that determine weed community dynamics. Temperature and water potential are two critical factors for germination [14, 39–41]. Therefore, predicting population establishment and development is particularly important for invasive species due to their devastating effects for ecological balance and human health. The germination capacity of *C*. *pauciflorus* under different temperatures and water potentials indicates the possibility of invasion of specific areas with similar climatic conditions. Thus, invasion risk evaluation is critical and exploring effective ways of inhibiting a prospective invasion is paramount.

Invasive weeds generally germinate in variable conditions. E.g. *Piper aduncum* has been reported to be able to germinate under constant temperatures from 15°C to 35°C [19]. *Iresine diffusa* has been reported to germinate best (> 80%) in all tested variable day and night temperatures (5/15°C, 10/20°C, 15/25°C, and 20/30°C), showing a germination potential for constant temperatures from 5°C to 30°C [38]. Our results indicate that *C*. *pauciflorus* seeds germinate within a broad range of alternating temperatures (10/20°C, 15/25°C, 18/28°C, 20/30°C, 25/35°C, and 30/40°C). Optimal germination occurred from 10/20°C to 30/40°C for M seeds and from 18/28°C to 30/40°C for P seeds (Fig 2 and Table 1), reflecting the ability of *C*. *pauciflorus* to germinate well in broad regions. This also suggests prospective germination from spring to autumn for temperate regions or throughout the whole year for tropical and subtropical regions. At our experimental site, soil temperature increased from 12°C (April) to 27°C (July) before dropping back to 9°C (October). Accompanying species (*Agriophyllum squarrosum* and *Artemisia halodendron*) typically germinate from mid-April or early-May [42], indicating no germination preference of *C*. *pauciflorus* compared to native species. Qu et al. [29] reported optimal germination performance of *C*. *pauciflorus* at 25°C, while maximal germination performances of other invasive weeds (*Piper aduncum* and *Iresine diffusa*) were observed at 25°C and 20°C, respectively [19, 38]. In our study, maximal germination was observed for 25/35°C and 20/30°C for M and P seeds, respectively. However, temperatures outside the optimum range (i.e. 5/15°C for M seeds and 5/15°C, 10/20°C and 15/25°C for P seeds) reduced germination percentages and prolonged the time until germination, thus decreasing germination of *C*. *pauciflorus* during the cold months or in cold regionsand minimizing their invasion capability accordingly.

Previous studies reported that *C*. *pauciflorus* can be outperformed with irrigation than without irrigation in biomass and many morphological characteristics such as height, seed number, tiller number, and leaf area [43, 44]. A further germination study using different level of humidity reported 6% as the minimum humidity for *C*. *pauciflorus* germination [29]. This indicates that *C*. *pauciflorus* is sensitive to the water condition during the whole growing season. Our results reveal that exposure to an increasing water potential substantially decreased germination percentage and extend germination time for *C*. *pauciflorus*. Similar results were obtained by Acosta et al. [38] in a germination study under different temperature, light and water potential treatments for *Iresine diffusa*: germination reduced significantly for an osmotic potential of -0.4 Mpa. *C*. *pauciflorus* in our experiment was sensitive to increasing osmotic stress. Its occurrence is rare under extreme drought conditions. Rainfall in Horqin is typically low during April and May [42], but still likely sufficient for *C*. *pauciflorus* germination. Moreover, *C*. *pauciflorus* germination was more sensitive at 10/20°C than at the optimal treatment (20/30°C), indicating *C*. *pauciflorus* is relatively vulnerable in spring and autumn, suggesting this period to be ideal to control further development.

Seed heteromorphism is an adaptation strategy of species to temporally or spatially variable habitats [45], seed type and size is a major influence for germination [22, 23, 46]. Brändel M [23] discovered different type of achenes of *Bidens frondosa* showed different dormancy and germination. Xu et al. [47] found seed size to pose a major influence on germination rate and percentage of *C*. *pauciflorus*. Correspondingly, in our experiment, quicker and improved germination was observed in M seeds compared to P seeds. The difference in mass might be one of the factors that contribute to the different germination behavior of M and P seeds. It represents the reproductive strategy of varying allocation of storage reserves within each seed, guaranteeing that at least one seed per bur germinates and contributes to successful seedling establishment the following season. Besides, P was more sensitive under both 10/20°C and 20/30°C conditions. Also, M and P seeds had different T_base_ and W_base_ (Fig 4), representing phenotypic plasticity to environmental stress. A lower base temperature was also observed with larger seeds in of *Eurotia lanata* [46]. These results support the hypothesis that temperature, water potential and seed type are all involved in regulating the germination of *C*. *pauciflorus*. Therefore, the prospective distribution and invasion capability of *C*. *pauciflorus* seems to mainly be dependent on water and temperature, which is in agreement with data for other weeds [19, 48]. Other factors such as soil type, and light in the natural grassland as well as fertilizer in the cropland need to be explored in the future to enable a more accurate evaluation for the prospective establishment of *C*. *pauciflorus*. Further studies need to be conducted to understand the effects of higher temperatures: the ceiling temperature treatment in our study did not detect the actual T ceiling.

Invasion success is a complicated process that involves interactions with the environment. Germination is the necessary prerequisite for establishment and development. *C*. *pauciflorus* could adapt to a broad temperature and performs with medium tolerance to water. These characteristics combined with the elongated spine on the surface of each bur greatly increase its adaptability and propagation. This may be one of the main reasons why it can easily invade a range of environments. The difference in seed size may be a reproductive strategy. This combined with the differences in T_base_ and W_base_ leads to the observed heteromorphism of seeds to ensure a successful establishment and seed production. The different response of M and P seeds to temperature and water appears to be an adaptation to the limited and unpredictable rainfall of the habitats occupied by this species. *C*. *pauciflorus* is well adapted to the semiarid subtropical or tropical systems and can germinate from spring to autumn or all the year round. It is hard to effectively control it if herbicide for already growing weed is also applied to only just germinating weeds in the soil. Therefore, both seed and seedling should be taken into account when applying herbicide.

The present report provides critical information on the effects of environmental factors (temperature and water potential) and seed type on seed germination as well as on possible mechanisms of *C*. *pauciflorus* invasion. Further research is still required to elucidate the mechanism of seed germination via comparing *C*. *pauciflorus* germination with native plant species at field scale. Germination variations of seed samples distributed in all different places should be conducted to further evaluate the stability and potential of invasion. Longer-term studies are needed to determine the impact of management methods and other climatic factors on the persistence of *C*. *pauciflorus* seed-banks. This vital information is required to develop efficient management strategies for this weed species.

## Supporting Information

S1 Fig Growth character of field *Cenchrus pauciflorus*. (A) seed, (B) seedling, (C) population, (D) spiking in the field, (E, F) spiking in different stages, (G) maturity, (H, I) whole single plant.
(TIF)

S2 Fig Measurement of M and P seeds of *Cenchrus pauciflorus*. Seed length, width and thickness are marked with line AB, line CD and line EF, respectively.
(TIF)

S1 Table Characteristic of M and P seeds of *Cenchrus pauciflorus*. Lowercase letter indicates significant difference between M and P seed at P< 0.05.
(DOCX)

## References

[1] Gritti E, Smith B, Sykes M. Vulnerability of mediterranean basin ecosystems to climate change and invasion by exotic plant species. J Biogeogr. 2006;33(1):145–57.

[2] Bradley BA, Wilcove DS, Oppenheimer M. Climate change increases risk of plant invasion in the eastern United States. Biol Invasions. 2010;12(6):1855–72.

[3] Weber E, Sun SG, Li B. Invasive alien plants in China: diversity and ecological insights. Biol Invasions. 2008;10(8):1411–29.

[4] Xu H, Ding H, Li M, Qiang S, Guo J, Han Z, et al The distribution and economic losses of alien species invasion to China. Biol Invasions. 2006;8(7):1495–500.

[5] Luo J, Cardina J. Germination patterns and implications for invasiveness in three *Taraxacum* (Asteraceae) species. Weed Res. 2012;52(2):112–21.

[6] Pyšek P, Richardson DM. Traits associated with invasiveness in alien plants: where do we stand? Biol Invasions: Springer; 2008 p. 97–125.

[7] Perglova I, Pergl J, Skalova H, Moravcova L, Jarošík V, Pyšek P. Differences in germination and seedling establishment of alien and native Impatiens species. Preslia. 2009;81(4):357–75.

[8] Schlaepfer DR, Glättli M, Fischer M, van Kleunen M. A multi‐species experiment in their native range indicates pre‐adaptation of invasive alien plant species. New Phytol. 2010;185(4):1087–99. 10.1111/j.1469-8137.2009.03114.x 19968796

[9] Wainwright CE, Cleland EE. Exotic species display greater germination plasticity and higher germination rates than native species across multiple cues. Biol Invasions. 2013;15(10):2253–64.

[10] Fenner M. Seed ecology: Springer; 1985 p. 87–102.

[11] Dürr C, Dickie J, Yang XY, Pritchard H. Ranges of critical temperature and water potential values for the germination of species worldwide: contribution to a seed trait database. Agric For Meteorol. 2015;200:222–32.

[12] Song W, Zhou W, Jin Z, Cao D, Joel D, Takeuchi Y, et al Germination response of *Orobanche* seeds subjected to conditioning temperature, water potential and growth regulator treatments. Weed Res. 2005;45(6):467–76.

[13] Schonbeck MW, Egley GH. Effects of temperature, water potential, and light on germination responses of redroot pigweed seeds to ethylene. Plant Physiol. 1980;65(6):1149–54. 16661350 10.1104/pp.65.6.1149 PMC440500

[14] Guillemin JP, Gardarin A, Granger S, Reibel C, Munier JN, Colbach N. Assessing potential germination period of weeds with base temperatures and base water potentials. Weed Res. 2013;53(1):76–87.

[15] Windauer LB, Martinez J, Rapoport D, Wassner D, Benech AR. Germination responses to temperature and water potential in *Jatropha curcas* seeds: a hydrotime model explains the difference between dormancy expression and dormancy induction at different incubation temperatures. Ann Bot. 2011:242. 10.1093/aob/mcr242 PMC3241573 21917817

[16] Hu XW, Fan Y, Baskin CC, Baskin JM, Wang YR. Comparison of the effects of temperature and water potential on seed germination of *Fabaceae* species from desert and subalpine grassland. Am J Bot. 2015;102(5):649–60. 10.3732/ajb.1400507 26022479

[17] Emmerich WE, Hardegree SP. Polyethylene glycol solution contact effects on seed germination. Agron J. 1990;82(6):1103–7.

[18] Ebrahimi E, Eslami SV. Effect of environmental factors on seed germination and seedling emergence of invasive *Ceratocarpus arenarius*. Weed Res. 2012;52(1):50–9.

[19] Wen B, Xue P, Zhang N, Yan Q, Ji M. Seed germination of the invasive species *Piper aduncum* as influenced by high temperature and water stress. Weed Res. 2015;55(2):155–62.

[20] Wen B. Effects of high temperature and water stress on seed germination of the invasive species Mexican sunflower. Plos One. 2015;10(10):e0141567 10.1371/journal.pone.0141567 26509675 PMC4624788

[21] Guillemin JP, Gardarin A, Granger S, Reibel C, Munier JN, Colbach N. Assessing potential germination period of weeds with base temperatures and base water potentials. Weed Res. 2013;53(1):76–87.

[22] Fumanal B, Chauvel B, Sabatier A, Bretagnolle F. Variability and cryptic heteromorphism of *Ambrosia artemisiifolia* seeds: what consequences for its invasion in France? Ann Bot. 2007;100(2):305–13. 10.1093/aob/mcm108 17575284 PMC2735321

[23] Brändel M. Dormancy and germination of heteromorphic achenes of *Bidens frondosa*. flora—morphology, distribution, Funct Ecol Plants. 2004;199(3):228–33.

[24] Imbert E, Escarré J, Lepart J. Seed heteromorphism in *Crepis sancta* (Asteraceae): performance of two morphs in different environments. Oikos. 1997:325–32.

[25] Gómez JM, Husband B. Bigger is not always better: conflicting selective pressures on seed size in *Quercus Ilex*. Evolution. 2004;58(1):71–80. 15058720 10.1111/j.0014-3820.2004.tb01574.x

[26] Fandrich L. Temperature effects on jointed goatgrass (*Aegilops cylindrica*) seed germination. Weed Sci. 2005;53:594–599.

[27] Quattrocchi U. CRC world dictionary of grasses: common names, scientific names, eponyms, synonyms, and etymology-3 volume set: CRC Press; 2006.

[28] Holm LRG. The world's worst weeds: distribution and biology: Krieger publishing company; 1991.

[29] Na C. Effect of four kinds of environment factors on seed germination of *Cenchrus pauciflorus* Benth. Seed. 2011;3:9.

[30] Du Gm, Cao FQ, Liu W, Hao FG, Liu BQ. The distribution and harmfulness of *Cenchrus Pauciflorus* Benth. in Liaoning province. Grassland of China. 1995.

[31] Parsons WT, Cuthbertson EG. Noxious weeds of Australia: CSIRO publishing; 2001.

[32] Mifsud S, Casha A. Two new alien grasses from sand dunes of Ghadira bay in Malta. Central Mediterranean Naturalist. 5(2):6–9.

[33] Dana E, Sanz EM, Sobrino E. Plant invaders in Spain [check-list]. 2001.

[34] Zhu M, Chen X, Han Z, Zhang G, Qu B. Study on leaf photosynthetic characteristics of field sandbur (*Cenchrus pauciflorus* Benth.) in China. Middle East J Sci Res. 2011;8(2):479–82.

[35] Zhao Y, Lv LY, Wang W, Han ZS, Lu Y, Luo XZ. Study on the effects of different row spacing of alfalfa on *Cenchrus pauciflorus* control. Pratacult Sci. 2010;4:21.

[36] Lv LY, Zhao Y, Wang HX, Wang W. Effects of mowing on plant regrowth and reproduction characteristics of invasive *Cenchrus pauciflorus*. Pratacult Sci. 2011;1:21.

[37] Trudgill D, Honek A, Li D, Van SN. Thermal time–concepts and utility. Ann Appl Biol. 2005;146(1):1–14.

[38] Acosta J, Bentivegna D, Panigo E, Dellaferrera I, Alisio M, Perreta M. Influence of environmental factors on seed germination and emergence of *Iresine diffusa*. Weed Res. 2014;54(6):584–92.

[39] Flores J, Briones O. Plant life-form and germination in a Mexican inter-tropical desert: effects of soil water potential and temperature. J Arid Environ. 2001;47(4):485–97.

[40] Windauer LB, Martinez J, Rapoport D, Wassner D, Benech AR. Germination responses to temperature and water potential in *Jatropha curcas* seeds: a hydrotime model explains the difference between dormancy expression and dormancy induction at different incubation temperatures. Ann Bot. 2012;109(1):265–73. 10.1093/aob/mcr242 21917817 PMC3241573

[41] Belo RG, Tognetti J, Benech AR, Izquierdo NG. Germination responses to temperature and water potential as affected by seed oil composition in sunflower. Ind Crop Prod. 2014;62:537–44.

[42] Cui JY, Li YL, Zhao HL, Su YZ, Drake S. Comparison of seed germination of *Agriophyllum squarrosum* (L.) Moq. and *Artemisia halodendron* Turcz. ex Bess, two dominant species of Horqin Desert, China. Arid Land Res Manag. 2007;21(3):165–79.

[43] Zhang ZX, Zhang K, Tian X. Characteristics of biological components of *Cenchrus pauciflorus* under wet and dry habitats. Pratacult Sci. 2012;12:21.

[44] Zhang ZX, Tian X. Characteristics of biomass allocation of *Cenchrus pauciflorus* under arid and irrigated habitats. Pratacult Sci. 2011;2:5.

[45] Venable DL, Dyreson E, Pinero D, Becerra JX. Seed morphometrics and adaptive geographic differentiation. Evolution. 1998:344–54. 28568325 10.1111/j.1558-5646.1998.tb01636.x

[46] Wang R, Bai Y, Tanino K. Effect of seed size and sub-zero imbibition-temperature on the thermal time model of winterfat (*Eurotia lanata* (Pursh) Moq.). Environ Exper Bot. 2004;51(3):183–97.

[47] Xu J, Li QF, Wang SY, Pang RY. Research on flowering behavior and seed germination of *Cenchrus pauciflorus*. Chinese J Grassland. 2011;2:5.

[48] Ebrahimi E, Eslami S. Effect of environmental factors on seed germination and seedling emergence of invasive *Ceratocarpus arenarius*. Weed Res. 2012;52(1):50–9.

