# Supplementary figures and images for: Field Sandbur (Cenchrus pauciflorus) Seeds in the Same Bur Respond Differently to Temperature and Water Potential in Relation to Germination in a Semi-Arid Environment, China
Source: PLoS One. 2016 Dec 19;11(12):e0168394. doi: 10.1371/journal.pone.0168394 (PMC5167391; doi:10.1371/journal.pone.0168394)

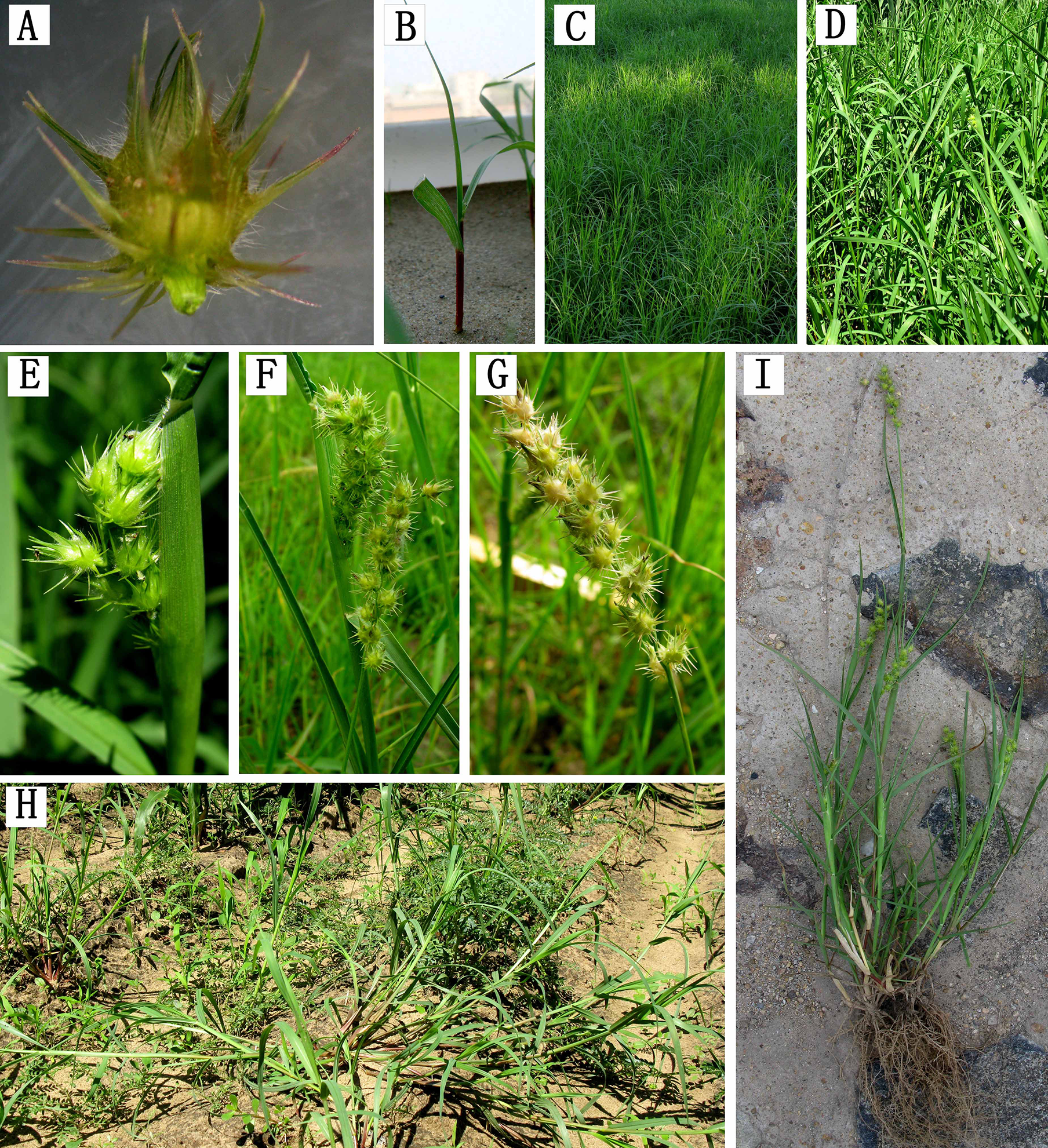

Supplement: S1 Fig — (A) seed, (B) seedling, (C) population, (D) spiking in the field, (E, F) spiking in different stages, (G) maturity, (H, I) whole single plant. (TIF) [file pone.0168394.s001.tif]

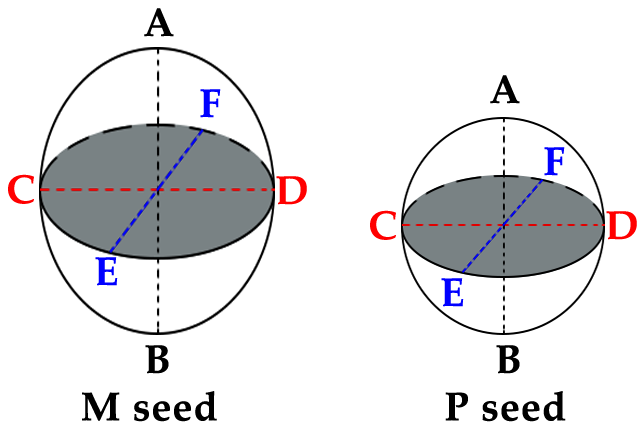

Supplement: S2 Fig — Seed length, width and thickness are marked with line AB, line CD and line EF, respectively. (TIF) [file pone.0168394.s002.tif]
