# Supplementary material for: Field Sandbur (Cenchrus pauciflorus) Seeds in the Same Bur Respond Differently to Temperature and Water Potential in Relation to Germination in a Semi-Arid Environment, China
Source: PLoS One. 2016 Dec 19;11(12):e0168394. doi: 10.1371/journal.pone.0168394 (PMC5167391; doi:10.1371/journal.pone.0168394)
Supplement: S1 Table — Lowercase letter indicates significant difference between M and P seed at P< 0.05. (DOCX) [file pone.0168394.s003.docx]

**S2 Table** Characteristic of M and P seeds of *Cenchrus pauciflorus*

| Indicator | Seed type | Value | SE |
| --- | --- | --- | --- |
| Seed weight (mg) | M | 7.72^a^ | (0.07) |
|  | P | 5.43^b^ | (0.06) |
| Seed length (mm) | M | 3.21^a^ | (0.01) |
|  | P | 2.69^b^ | (0.01) |
| Seed width (mm) | M | 2.48^a^ | (0.01) |
|  | P | 2.35^b^ | (0.01) |
| Seed thickness (mm) | M | 1.4^a^ | (0.02) |
|  | P | 1.2^b^ | (0.02) |

Note: Lowercase letter indicates significant difference between M and P seed at P< 0.05.
